# Evaluating methods for estimating home ranges using GPS collars: A comparison using proboscis monkeys (*Nasalis larvatus*)

**DOI:** 10.1371/journal.pone.0174891

**Published:** 2017-03-31

**Authors:** Danica J. Stark, Ian P. Vaughan, Diana A. Ramirez Saldivar, Senthilvel K. S. S. Nathan, Benoit Goossens

**Affiliations:** 1 Organisms and Environment Division, Cardiff School of Biosciences, Cardiff University, Sir Martin Evans Building, Museum Avenue, Cardiff, United Kingdom; 2 Danau Girang Field Centre, c/o Sabah Wildlife Department, Wisma Muis, Kota Kinabalu, Sabah, Malaysia; 3 Sabah Wildlife Department, Wisma Muis, 88100 Kota Kinabalu, Sabah, Malaysia; 4 Sustainable Places Research Institute, Cardiff University, 33 Park Place, Cardiff, United Kingdom; Sichuan University, CHINA

## Abstract

The development of GPS tags for tracking wildlife has revolutionised the study of home ranges, habitat use and behaviour. Concomitantly, there have been rapid developments in methods for estimating habitat use from GPS data. In combination, these changes can cause challenges in choosing the best methods for estimating home ranges. In primatology, this issue has received little attention, as there have been few GPS collar-based studies to date. However, as advancing technology is making collaring studies more feasible, there is a need for the analysis to advance alongside the technology. Here, using a high quality GPS collaring data set from 10 proboscis monkeys (*Nasalis larvatus*), we aimed to: 1) compare home range estimates from the most commonly used method in primatology, the grid-cell method, with three recent methods designed for large and/or temporally correlated GPS data sets; 2) evaluate how well these methods identify known physical barriers (e.g. rivers); and 3) test the robustness of the different methods to data containing either less frequent or random losses of GPS fixes. Biased random bridges had the best overall performance, combining a high level of agreement between the raw data and estimated utilisation distribution with a relatively low sensitivity to reduced fixed frequency or loss of data. It estimated the home range of proboscis monkeys to be 24–165 ha (mean 80.89 ha). The grid-cell method and approaches based on local convex hulls had some advantages including simplicity and excellent barrier identification, respectively, but lower overall performance. With the most suitable model, or combination of models, it is possible to understand more fully the patterns, causes, and potential consequences that disturbances could have on an animal, and accordingly be used to assist in the management and restoration of degraded landscapes.

## Introduction

The development of global positioning system (GPS) tags for tracking wildlife has revolutionised the study of home ranges, habitat use and behaviour [1]. GPS telemetry has provided the opportunity to simultaneously track multiple animals with improved locational accuracy, without the limitations associated with radio tracking or direct human observation, such as biases due to bad weather, length of time followed, distance covered, or difficult terrain [1,2]. However, the larger data sets and more frequent sampling intervals compared to traditional tracking technologies challenge the validity and feasibility of established data analysis methods, stimulating the development of new methods to reveal movement patterns, behaviour and estimate home ranges [3,4]. These are important developments, yet relatively little guidance is available to help researchers choose between them.

Home range estimation is one of the main applications of GPS tagging data [4]. An animal’s home range is traditionally defined as the area used for feeding, sleeping, finding mates, and raising young [5], but more modern definitions describe it in terms of the area across which an animal has a defined probability of occurrence during a specified time window [6]. Furthermore, the home range is suggested to be part of the animal’s cognitive map, in which movements are planned based on the nutritional state or motivation of the animal [7]. The cognitive map of an animal may also include areas which it is aware of but does not go to, due to smell, sight or hearing [7]. Within the home range, important information for ecology and conservation includes the total area required by the study subjects, the time spent in different areas and how frequently different parts are used [8]. This is often displayed in terms of a utilisation distribution (UD), which is the relative frequency at which an animal uses different parts of its home range [9,10]. This in turn can help to identify the core area where an animal spends most of its time, including important feeding and resting sites [5,11,12]. Characterising these different aspects of home ranges, and understanding the processes of habitat selection, movement and activity patterns and how they respond to environmental and anthropogenic changes, are all important for the conservation management of wild populations [11,13,14].

Since the first use of radio-collaring for studying home ranging in the 1960’s [15], methods for analysing tracking data have evolved continuously, accelerating after 2000 with an end to blocking GPS accuracy and rapid technological developments [16]. Home range estimators vary widely in their sophistication, assumptions and the level of detail revealed, but fall into two main groups: location-based methods, which ignore temporal information and include many of the traditional methods of analysis, and movement based methods, which are more recent developments and combine time and location data. Both categories include methods for estimating utilisation distributions.

Location-based estimators tend to be conceptually simple and computationally efficient. The grid-cell method (GCM) is the simplest approach to estimating the utilisation distribution, in which a grid is superimposed over the area, and the number of times an animal enters each cell counted [17,18]. Other approaches based upon parametric kernel density estimators are also used (e.g. [19–22]). Although GCM is useful in showing hot spots in utilisation patterns, its main disadvantage is in measuring overall home range size, as well as estimating range boundaries, i.e. barriers or ranges with complex boundaries [18]. Both GCM and parametric kernels are widely used throughout ecological studies, but the disadvantage of these approaches is that they are sensitive to the degree of smoothing (e.g. grid cell size or kernel widths) [4]. These approaches also struggle in habitats with barriers to movement or where there are abrupt changes in habitat type [23]. In common with most location-based parametric methods, they also assume that points are independent from each other—an assumption that is rarely met by the short time intervals between GPS fixes [24].

In response to the limitation of parametric methods in handling barriers or habitat edges and assumptions requiring GPS points to be independent, the local convex hull nonparametric kernel method (LoCoH) was developed [23]. LoCoH calculates the convex hull around each GPS fix based upon its close neighbours, before forming density isopleths by merging hulls together [23,25]. Neighbours can be defined in different ways, leading to different versions of LoCoH [25]. Unlike parametric kernel methods, LoCoHs do not require the user to make any pre-assumptions of the functional form for the kernels, and therefore they are more successful at identifying the true boundaries as the density of data increases [25].

Temporal autocorrelation between fixes has traditionally been considered a problem in home range analysis, often leading to large amounts of data being discarded to produce ‘independent’ observations [26,27]. By contrast, movement-based density estimates combine the location and time of a fix, as well as being able to incorporate activity data collected between fixes by the movement sensors built into most GPS collars [28–30]. Two of the main methods are adaptive time LoCoH (T-LoCoH), which adds temporal information to the basic LoCoH analysis whilst retaining the desirable edge-detection qualities [30], and biased random bridges (BRB), a development on kernel density estimation that combines serially correlated GPS fixes with high frequency activity data to estimate fine scale movements and habitat use [24,28,31]. Recognising the value of accurately resolving range edges, BRB allows barriers to movement (e.g. rivers) to be specified, further reducing biases associated with parametric kernel smoothing [28].

The number of different home range analysis methods that are available, combined with the rapid rate of development of these analyses, can make it difficult for researchers to choose between methods. Whilst the research question should be the primary driver of the method selected [8], a greater understanding of how different methods perform would aid this selection and assist comparisons amongst existing home range estimates. Within the field of conservation biology, there has been an increase in studies comparing different home range estimators with GPS collaring data (e.g. [25,32–34]). In primatology, however, this issue has received scant consideration as home range studies are still in their infancy, with few GPS-based studies and the analysis often relying upon the GCM (e.g. [12,17,18,35]). Here, using a high quality GPS collaring data set collected from 10 proboscis monkeys (*Nasalis larvatus*) in northern Borneo, we aimed to: 1) compare home range estimates generated by the most commonly used estimator in primatology, the GCM, with three alternative methods designed for large and/or temporally correlated data sets (adaptive LoCoH, time LoCoH and BRB); 2) evaluate model performance with known physical barriers for a species which recurrently utilises forest edges; and 3) test which of the models is the most versatile and robust by simulating less intense sampling regimes resulting from technological limitations or failures.

## Methods

### Ethics statement

All animal handling was carried out in accordance with the current laws of Malaysia and Sabah Wildlife Department’s Standard Operation Procedures on Animal Capture, Anaesthesia and Welfare. Permission was granted by Sabah Biodiversity Centre (permit JKM/MBS.1000-2/2 JLD.3 (73)). The work carried out during this study was in accordance with the Weatherall report, and followed the guidelines for non-human primates as described by Unwin et al. [36]. All efforts were made to ensure the welfare, and reduce stress of the animals, with the addition of full personal protective equipment worn by all team members throughout the process to prevent human-primate disease transmission. A veterinarian specialised in the capture and anaesthesia of wildlife performed the darting, having previously conducted an evaluation of the area and target individual to minimise risk to the animals. Animals were anaesthetised using Zoletil 100 (Tiletamine + Zolazepam; 6–10 mg/kg), and a prophylactic dose of Alamycine LA (20 mg/kg) and Ivermectine (0.2 mg/kg) was given as a preventative measure to assist in the post-anesthesia recovery. Anaesthesia and the vital signs were monitored throughout the procedure.

### Study site and subjects

This study took place in the Lower Kinabatangan Floodplain, Sabah, Malaysian Borneo (5°18’N—5°42’N and 117°54’E—118°33’E). The floodplain consists of 420 km^2^ of protected forest and approximately 100 km^2^ of state and private forest, and is a mosaic of agricultural land and natural forest types, including dry lowland forest, semi-inundated, semi-swamp/grassy forests and swamp [37,38].

**Fig 1:**
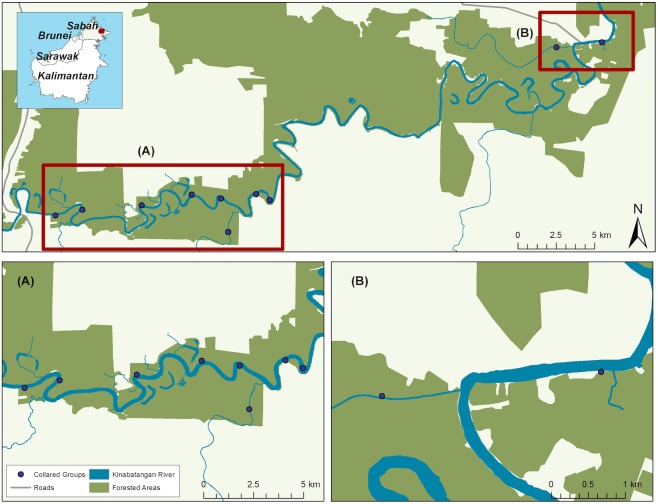
Collaring sites of 10 proboscis monkeys along the Kinabatangan River, Sabah, Malaysia.

Ten proboscis monkeys were collared from different one-male social units spread along the Lower Kinabatangan River, covering a range of habitat quality, and forest fragment sizes (Fig 1). Proboscis monkeys travel as an integrated unit, so the movement of a single individual can be considered to represent the whole group [39]. Collaring locations were always >2 km apart, or on opposite sides of the river, to minimise potential overlap between home ranges. Eight individuals were collared within protected forest, and two were collared in unprotected forests that connect protected forest lots. GPS collars were fitted to six males and four females (male: Lotek Biotrack GSM WildCellSD; female: e-obs UHF 1C-Light) by a qualified veterinarian, and weighed <2% of the individual’s body mass (cf. recommended 5% maximum [40]. Collars were fitted in 2011–2014 and provided data for 109–401 days (S1 Table). By equipping the male’s collars with a pre-programmed automatic release mechanism after 12 or 18 months, and the female collars with leather spacers (due to weight constraints), no recapture was necessary. To maximise battery life, collars were programmed to record hourly positions between 0500hr and 1900hr (at least 30 minutes before sunrise/after sunset), as proboscis monkeys are sedentary after dark [41].

### Home ranging data sets

To improve the quality of the data set as per Bj**ø**rneraas [42] the GPS data were filtered prior to analysis to remove locations which were: i) fixed by fewer than four satellites; or ii) further from both the previous point and subsequent point than an animal is able to travel in the elapsed time. This distance was calculated using extensive ground follows of a single proboscis monkey group from a previous study [43] that calculated the estimated daily path length during ground follows to be 799 m, Therefore any distance greater than half that between consecutive hourly points was excluded to account for GPS error. To account for pseudo-replication in the home range estimates due to the 1900hr and subsequent 0500hr fix being taken in the same tree, all 0500hr points were removed. After the data screening, only 8.3% of points were removed. Compared to other studies, which have had to remove 16–26% of their points [44,45], this study only rejected a small proportion of points, and is therefore considered a high quality data set.

Many GPS collaring studies have a lower fix frequency than in the current study (e.g. 4h interval) to maximise battery longevity when tag weight is restricted by the study species [46]. In addition, a large proportion of GPS fixes often fail (e.g. <60% fix success rate [47]) or are rejected due to low quality, based on high dilution of precision values [16,42,44]. To investigate the effects of these two factors on home range estimation, we compared home range estimates using the complete data set to those based on two subsets of the data that simulated lower fix frequency or higher fix error rates [18]. Simulation 1 removed 75% of the data to create a regular interval of four hours between fixes to mimic the reality that many GPS tracking studies have to take less frequent fixes in order to elongate the total collaring study period. Simulation 2 represented the situation where fixes were not always possible, or the GPS error was too high for the fix to be usable. This is more prevalent for smaller collars or animals living on the forest floor [32,47–49], and results in irregular time intervals. As it is possible for multiple fixes to fail in a day, but rarely that all scheduled fixes would fail, a minimum of five fixes were randomly selected each day, with the maximum potential for 14 hourly fixes, to represent fix failure (S1 Table).

### Home range estimation

Utilisation distributions were estimated using four approaches: i) GCM, ii) adaptive localised convex hull (*a*-LoCoH), iii) time-based adaptive localised convex hull (T-LoCoH), and iv) BRB. GCM and *a*-LoCoH are location-based estimators, whereas T-LoCoH and BRB incorporate time i.e. are movement-based. GCM was calculated in Geospatial Modelling Environment [50]. The remaining estimators were calculated in R 3.1.3 [51] using the packages *adehabitatHR*, *adehabitatLT* (*a*-LoCoH and BRB) [52,53] and *tlocoh* [30]. The UDs were based on the 90 percentile for overall home range size and 50% for the core area [54].

GCM used a grid with 50 x 50 m cells, consistent with previous proboscis monkey studies [43,55]. Despite the recommendation to exclude a proportion of outlying points, as they often represent imprecisions in location estimates or exploratory movements rather than points within the functional home range, most studies continue to use 100% of the points for GCM home range estimates. To reduce the bias in home range estimations that include imprecise or exploratory movements, as well as to make the GCM method comparable to the other methods examined in this study, the least dense 10% and 50% cells were eliminated for the estimates of total and core UD, respectively.

*a*-LoCoH is a development of the traditional minimum convex polygon method for calculating home ranges [25]. It calculates a convex hull for every point in the data set, based on its nearest neighbours, before merging the hulls into a set of nonparametric kernels based on the density of points [25]. The nearest neighbours for each GPS fix are the sets of points whose cumulative distance to the focal fix are less than or equal to a defined threshold, *a*, resulting in areas of higher use having smaller convex hulls [25]. The value of *a* was selected using the two-part method recommended by Getz et al. [25]: i) using the maximum distance between two GPS fixes in the data set as the starting value for *a*, before ii) being further refined by rounding to the nearest multiple of 10 by visually assessing the maps using the “minimum spurious hole covering” technique, which ensures the physical features that cannot form part of the home range (e.g. lakes) are excluded from the *a*-LoCoH estimate [25,30] (S2 Table).

Adaptive T-LoCoH builds upon *a*-LoCoH by incorporating time into the model. A time-scaled distance factor is used to select nearest neighbours for T-LoCoH by calculating the maximum theoretical velocity of an individual [30]. The scaling factor, *s*, specifies the maximum amount of time at which spatially neighbouring, but not necessarily sequential, GPS fixes are still considered to be temporally correlated to the focal location, and therefore included as a nearest neighbour [30]. By increasing *s*, time becomes more important in defining the degree of correlation in the distance between fixes and the time between those fixes [32]; when *s* = 0, time is not considered [30]. Lyons et al. [30] recommend that the value of *s* should ensure that 40–60% of hulls are constructed using temporally correlated fixes, so that both the spatial and temporal data are being considered relatively equal in the analysis; we used 50% throughout for consistency. The *a*-value was then selected using the MHSC technique (S2 Table).

BRB is a movement-based kernel method that links successive GPS fixes and then interpolates between them to develop a smoothed kernel density estimate for each interpolated location [32]. To interpolate between locations, BRB assumes that the animal is moving towards the next location, but incorporating a random component to model deviations from the straight line path [56]. BRB requires three main parameter values to be set based on biological or technological knowledge. The maximum time threshold (*T*_*ma*x_) is the longest period between points before they are no longer considered to be autocorrelated. Autocorrelation was determined by comparing the summed squared differences in step length between successive fixes with randomly permuted values of step length [57,58]. For the complete data set and Simulation 2, *T*_*max*_ = 7,800 sec (2 hours plus 10 minutes tolerance), and 29,400 sec for Simulation 1 (8 hours plus 10 minutes tolerance) [28]. The second parameter is the minimum step length (*L*_*min*_), which defines a distance between successive points below which the animal is considered stationary (e.g. when feeding or resting; [59]). To account for the possibility of an animal moving within a tree when foraging or due to social displacements, or possible false movements due to GPS error, which averaged 14.3 m (based on static collar tests with the collars set at a fixed location), track segments less than 15 m were assumed to be resting points (*L*_*min*_ = 15). Finally, the minimum smoothing parameter (*h*_*min*_) corresponds to the minimum standard deviation in relocation uncertainty [56]. It must be large enough to encompass the range of potential locations an animal could actually occupy whilst being recorded at the same point, while being less than half the mean distance travelled for the time *T*_*max*_ [28]. To assist in the selection of *h*_*min*_, the mean cosine of turning angles was calculated to estimate the tortuosity of the animal’s path, and thereby the uncertainty of a location between two recorded locations [60]. The mean cosine of turning angles in the tracking data was 0.30, suggesting an intermediate value of *h*_*min*_ between our observed standard deviation of relocation uncertainty (19.0 m) and half the mean distance travelled for time *T*_*max*_ (68.39 m). However, as boundary segment lengths must be greater than 3×Hmin or cannot be sharper than 90° [61], and the boundaries in this study had many sharp and tight bends, *h*_*min*_ was set at 25 m to account for these restrictions. See Benhamou [60] for full details of this process.

### Model comparisons and statistical analysis

The home range estimates produced by the four methods were compared in two ways: i) the overall dissimilarity between the utilisation distributions, and ii) specific characteristics of the range estimates (e.g. area). Overall dissimilarity was assessed by calculating the Hellinger distance between each pair of home range estimates and ordinating the resulting distance matrix using principal coordinate analysis (PCoA) [62]. All 40 home range estimates (10 individuals x four methods) were converted to rasters on a standard grid (identical coordinate origin and resolution) allowing pixel-by-pixel comparisons based on the row and column pairs between the paired maps [62]. The significance of apparent differences between the methods was tested using permutational multivariate analysis of variance (perMANOVA), using the *adonis* function in the *vegan* package [63,64]. Permutations were stratified by individual proboscis monkeys to control for individual differences and focus on the differences between the methods.

Five characteristics were used to compare home range estimates from the four methods in greater detail (Table 1). Although an animal’s ‘true’ home range is unknown using empirical data (as opposed to *in silico* comparisons), we can assess the *relative* properties of different estimators using a range of measures. Area, boundary complexity and patchiness provided information about the basic shape of the home range. Variation in these three properties can illustrate the likelihood of estimators under- or over-fitting, and therefore can be indicative of the models’ tendency to under- or overestimate of home range area, respectively. The complexity of a boundary can be used as a proxy to measure the relative goodness of fit of a home range, and may show that, due to irregular or concave boundaries created, it not only excludes areas which were not used, but also used areas [44]. Although patches in a home range may be indicative of differences in habitat quality [65] or an increase in speed through disfavoured areas to reach favoured areas, a large degree of patchiness may also mean that the pathways taken to the patches are not included due to the over-fitting. Accurate barrier detection is important for reliably delineating the edges of the home range. Methods that are unable to intrinsically delineate the edges of an animal’s range are particularly susceptible to boundary bias, particularly for quadrats that are in direct contact with the boundary, as values will be over- or under-estimated, depending on whether the quadrat lies on the unused side of the boundary, or the used side, respectively [28]. The area-under-the-curve (AUC) is a metric that has recently been used to determine the most appropriate home range estimator by assessing how well GPS fixes fit the contours of each estimator [66]. AUC values measured each home range estimator’s ability to discriminate between areas that had GPS fixes and those that did not [66]. In effect it provided a measure of accuracy–the agreement between the observed GPS points and the modelled utilisation distribution. The AUC value ranges between 0.5 to 1.0, with 0.5 equivalent to chance–no agreement between observed and modelled data–and a value of 1.0 indicating perfect agreement between the points and the utilisation distribution [67].

**Table 1:** Summary and methods used to calculate the physical characteristics used to compare the home range estimators.

Home range characteristics	Justification and method
Total home range area	Calculated in ArcGIS in ha
Boundary complexity	Edge density (ED) ratio: ED = perimeter (m) / area (ha) [68]. Higher numbers indicate more complex boundaries, which in turn can be used as a proxy for how the data fit the model (i.e. by creating irregular or concave boundaries [44]).
Patchiness	The number of separate patches. Being too patchy may indicate the model over-fitting (underestimating) the data and therefore not being as truly representative towards the area actually required by the animal [18].
Barrier detection	The percentage of the estimated home range that overlapped features known to be barriers to proboscis monkeys: large water bodies (main river and oxbow lakes) in this study. Small tributaries (<10 m wide) were not considered as true barriers, as proboscis monkeys are able to cross them easily [43]. The presence of water was determined using pre-existing drone imagery of the study area.
Area-under-the-curve (AUC)	The AUC is a measure of accuracy used to determine the most appropriate home range estimator by assessing how well GPS fixes fit the contours of each estimator, calculated using the *caTools* package in R based on [66].

Differences in the five home range descriptors between the four methods were tested using General Linear Mixed Models (GLMMs) using R’s *lme4* library [69], with estimation method as a fixed effect. The individual identification for each collared monkey was treated as a random effect in the models to account for multiple estimates of each individual’s home range, whilst sex was included as a covariate to control for differences between males and females. Significance of terms was tested with a likelihood ratio test, comparing nested models with and without the fixed effect of interest, and Tukey tests were carried out using the *multcomp* library to examine pairwise differences between the four methods [70].

The effects of reduced data quantity (Simulation 1 & 2) were assessed in two ways. First, a subsample overlap analysis was used to compare the home ranges estimated using the full data with those from the two simulations [18]. The percentage of the fixes from the full data set included within the subsampled home range was calculated for both simulations, as well as the percentage of area overlap between the complete and simulation ranges of the same method. A higher percentage of overlap and greater inclusion of fixes indicated a more robust model [18]. The second approach compared home range estimates from both simulations to the estimates obtained using the full data based on the five measures (Table 1). GLMMs were used, with fixed effects for data set (complete, Simulation 1 or Simulation 2) and estimation method, and a random effect for proboscis monkey individuals.

## Results

### Method comparisons using the full data set

**Fig 2:**
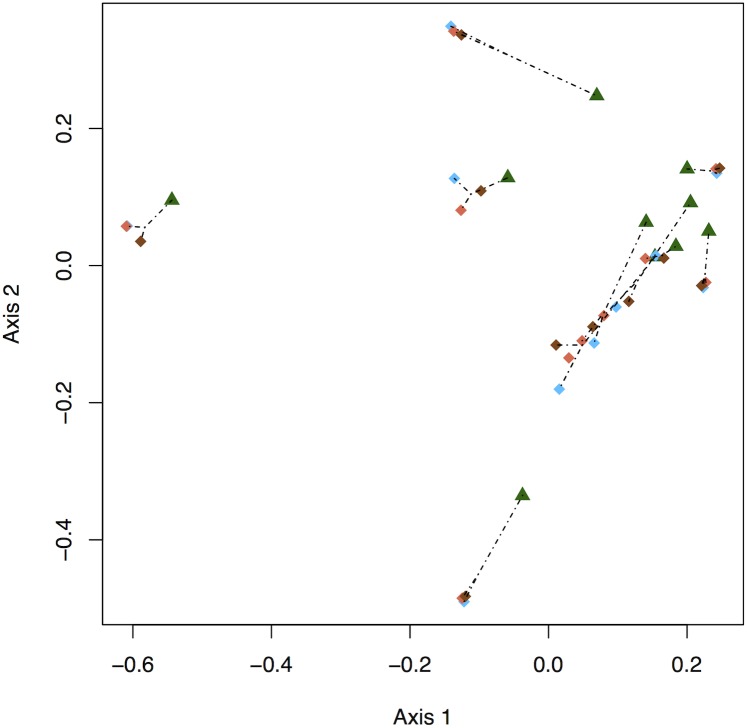
Principal coordinates plot of the home range estimators for 10 individual proboscis monkeys. Dotted lines indicate Hellinger distance, showing the differences between the ranges produced by four home range estimators (GCM, green triangle; a-LoCoH, blue; T-LoCoH, orange; and BRB, brown).

**Fig 3:**
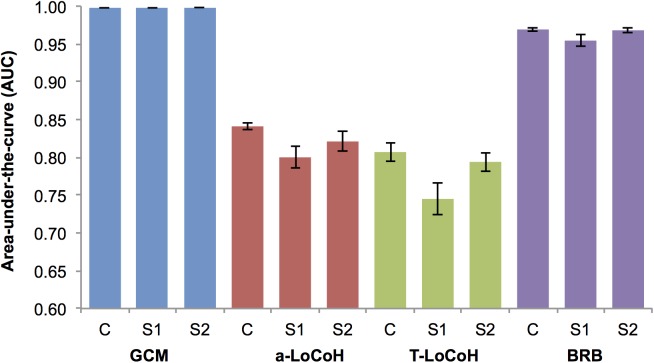
Mean (±SE) area-under-the-curve for the home range estimators (N = 10 individuals). Grid-cell method (GCM—blue), adaptive local convex hull (*a*-LoCoH—red), adaptive time local convex hull (T-LoCoH—green) and biased random bridges (BRB—purple), using the complete data set (C) and the simulated scenarios, with a decreased sampling interval (S1 = fixes every 4 hours), and simulating random failures (S2).

Utilisation distributions differed significantly among home range estimators (F(3,36) = 0.45, p = 0.001) and in how closely they matched the original GPS fixes (AUC; Chi-sq = 112.92, df = 3, p<0.001). GCM utilisation distributions were clearly separated from the other three methods, which were usually very similar to one another: *a*-LoCoH and T-LoCoH models were generally concordant, whilst half of the BRB models overlapped the LoCoH models, and the remaining half were still closer to the LoCoH methods than to the GCMs (Fig 2). GCM estimates agreed most closely with the raw GPS fixes (AUC = 0.998), followed by BRB (0.969), with the two LoCoH methods showing weaker agreement: *a*-LoCoH (0.841) and T-LoCoH (AUC = 0.807) (Fig 3). All pairwise comparisons of AUC were significantly different (p<0.05).

**Fig 4:**
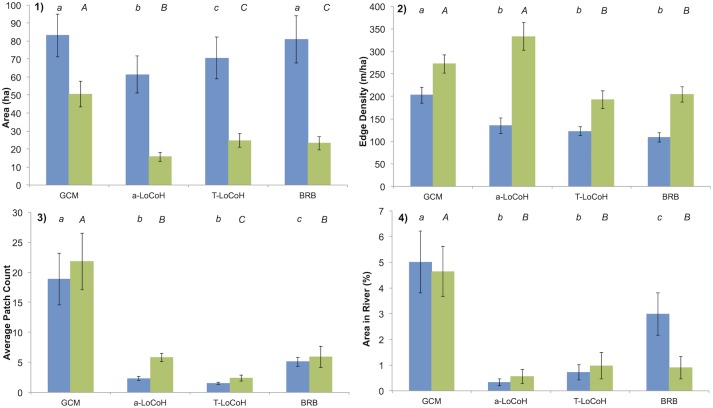
Summary of averages for overall (90%, blue) and core (50%, green) home range comparison variables (N = 10 individuals). (1) home range area; (2) boundary complexity (edge density); (3) patchiness and (4) barrier detection for: Grid-cell method (GCM), adaptive local convex hull (*a*-LoCoH), adaptive time local convex hull (T-LoCoH). a,b,c Pair-wise results from Tukey test; results significantly different from another (p<0.05) are indicated by a different letter, those with the same letter showed no significant difference. Lower-case letters represent overall home range differences, and upper-case letters represent core-range differences.

**Fig 5:**
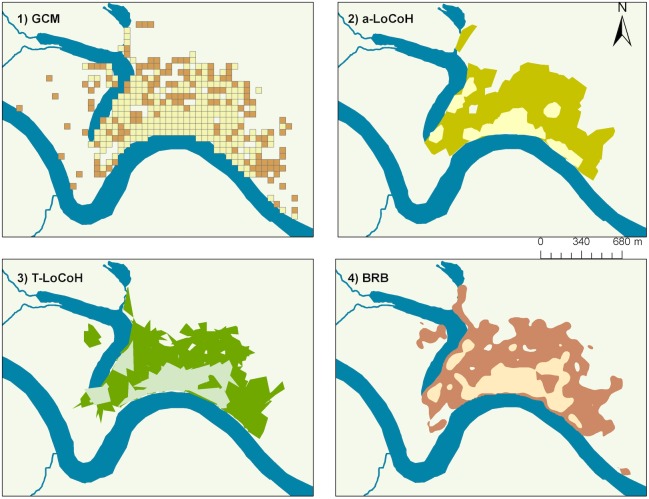
An example of the home range estimates produced for one proboscis monkey. Home range estimator (1) Grid-cell method (GCM), (2) adaptive local convex hull (*a*-LoCoH), (3) adaptive time local convex hull (T-LoCoH), and (4) biased random bridges (BRB); light colours = 50% isopleth, and dark colours = 90% isopleth.

The choice of home range estimate method also significantly affected the area, boundary complexity, patchiness and edge detection accuracy of the resulting home range estimates (all p<0.001; Fig 4, see S3 Table for detailed values and test statistics of overall and core range). GCM produced the largest, most patchy estimates, with the longest boundaries relative to area and the largest overlaps with the rivers/oxbow lakes for overall home range and core range (Fig 5). It differed significantly (all Tukey tests p<0.05) from all other methods on these four measures, with the exception of BRB for total area and *a*-LoCoH for boundary complexity of the core range. Using 100% of the points for GCM (as is commonly used in other studies), resulted in a home range estimate which was 22.06% larger, from 83.05 ha (range 35.00–167.25 ha) to 108.13 ha (range 41.25–217 ha; S4 Table).

a-LoCoH produced the smallest home range estimates, and was not significantly different than T-LoCoH in producing the least patchy estimates, with the least amount of overlap with the river and oxbow lakes. There was no difference in edge density between *a*-LoCoH, T-LoCoH and BRB in overall home range, but the core range edge density for *a*-LoCoH was significantly higher than that of T-LoCoH and BRB. BRB produced mid-range estimates for patchiness and barrier detection for the overall range, but was no different than *a*-LoCoH and T-LoCoH in its overlaps with rivers and oxbow lakes for core ranges

### Simulations

**Fig 6:**
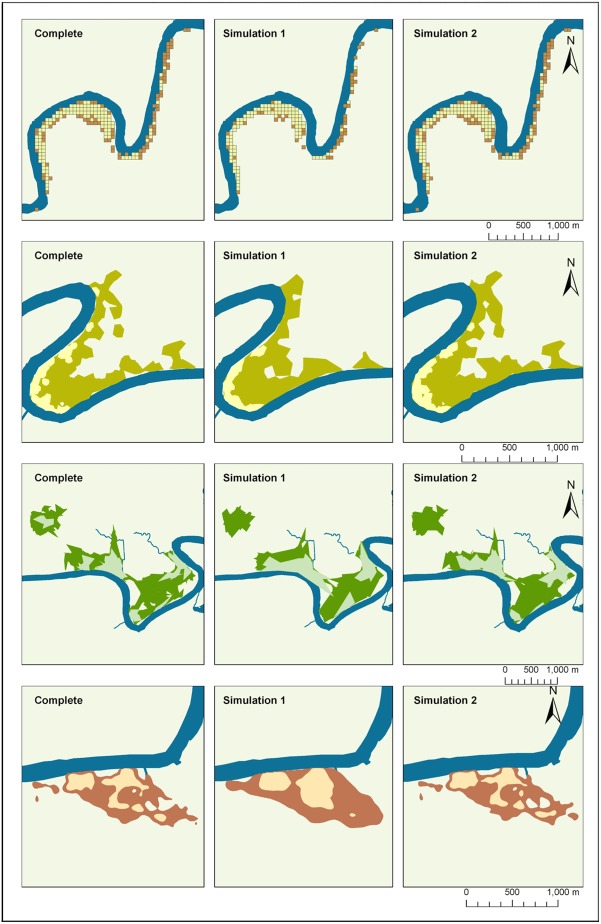
An example of selected home range estimators under different simulations. (A) grid-cell method (GCM), (B) adaptive local convex hull (*a*-LoCoH), (C) adaptive time local convex hull (T-LoCoH), and (D) biased random bridges (BRB). Simulation 1 simulated low fix rate (every 4 hours) and Simulation 2 simulated fix failures (light = 50% isopleth, and dark = 90% isopleth).

In the majority of cases, rarefaction of the GPS data (Simulation 1) or random removal of 5–14 points per day (Simulation 2) did not have significant effects upon the average characteristics of estimated UDs (Table 2, S5 Table). Where differences were detected, they occurred most frequently between Simulation 1 and the full data, and affected GCM and BRB to a greater extent than the two LoCoH techniques. GCM was the only method to experience a significant change in the sub-sample overlap analysis, with Simulation 1 having the lowest percentage of overlap. Despite changes in area and outline, the AUC values for GCMs and BRBs showed no difference between simulations, whereas this was the only measure by which *a*-LoCoHs and T-LoCoHs were affected, both producing smaller AUC values for Simulation 1 (Fig 3). Different methods responded to the simulations in different ways. For GCM, Simulation 1 produced smaller UDs, with more complex outlines, whereas areas from BRB estimates increased by approximately a factor of 1.4 (core) and 1.5 (overall), and had smoother boundaries. Simulation 2 did not differ from the full data for either measure. Patchiness only changed for BRB with Simulation 1, decreasing the number of patches more by a factor of 2.6 (core) to 2.7 (overall) from the complete model. The area overlapping the river was not significantly affected by either simulation, despite the significant changes in home range area and boundary complexity for GCMs and BRBs. (Fig 6).

**Table 2:** Summary of simulation home range models.

Simulation	Ave. Area (ha)	Ave. Edge Density (m/ha)	Ave. Patch Count	Area in river (%)	Point Inclusion (%)	AUC
*GCM*: *Complete*	83.05^a^	*202*.*77*^a^	*18*.*90*	*5*.*00*	*96*.*30*^a^	*0*.*998*
Simulation 1	40.73^b^	404.22^b^	34.90	6.34	85.53^b^	0.997
Simulation 2	71.15^a^	252.83^a^	24.10	4.64	94.28^a^	0.998
*a-LoCoH*: *Complete*	*61*.*41*	*134*.*79*	*2*.*30*	*0*.*33*	*89*.*78*	*0*.*841*^a^
Simulation 1	59.02	127.08	1.80	0.52	89.45	0.800^b^
Simulation 2	62.31	123.66	2.20	0.21	89.54	0.821^a^^,^^b^
*T-LoCoH*: *Complete*	*70*.*51*	*122*.*46*	*1*.*50*	*0*.*72*	*89*.*70*	*0*.*807*^a^
Simulation 1	73.78	99.16	1.20	2.95	90.16	0.745^b^
Simulation 2	72.93	108.63	1.60	1.20	89.85	0.794^a^
*BRB*: *Complete*	*80*.*89*^a^	*108*.*99*^a^	*5*.*10*^a^	*2*.*98*	*93*.*88*	*0*.*969*
Simulation 1	122.53^b^	60.38^b^	1.90^b^	6.71	97.34	0.954
Simulation 2	81.25^a^	111.08^a^	5.50^a^	3.04	93.98	0.968
Chi-sq value*	123.24	205.73	213.05	111.56	98.01	310.11

Grid-cell method (GCM), adaptive local convex hull (*a*-LoCoH), adaptive time local convex hull (T-LoCoH) and biased random bridges (BRB). Simulation 1 simulated low fix rate (every 4 hours) and Simulation 2 simulated fix failures. (S6 Table for core range model results)

a,b: Pair-wise results from Tukey test; results significantly different from another (p<0.05) are indicated by a different letter, those with the same letter showed no significant difference

*Chi-square values for GLMM likelihood ratio test: for all tests, df = 11 and p <0.001.

## Discussion

Considering the advances in methods for home range estimation over recent years, there have been relatively few studies examining the suitability of new methods for primate ecology or conservation [but see 17,63]. Here, we compared the most widely used approach in primatology (GCM) against several recently developed methods. This showed that the home range estimates produced by GCM were distinctly dissimilar from the others, even when only using 90% of the points instead of the standard 100% for GCM. For the physical characteristics, both LoCoHs were particularly robust to variations in sampling intensity, and were the best methods at detecting barriers. Next to GCM, BRB estimates agreed most closely with the raw data, even when sampling intensity varied. Despite BRBs similarity to GCM in terms of area and AUC values, the utilisation distributions were similar to the LoCoH methods, and produced intermediate results between GCM and LoCoHs. The results demonstrate that the choice of home range estimator can have important impacts on the conclusions drawn from a study, and could be important considerations in selecting a method for home range estimation (Table 3). We first consider some of the limitations to our study, before discussing the results in greater detail and concluding with some recommendations for future studies.

**Table 3:** Summary of the strengths and weaknesses of the home range estimators examined in this study.

Method	Strengths	Weaknesses	Requirements	Suitability
GCM	• Comparable to other studies • Identifies areas of importance • High AUC	• Sensitive to sample size • Cannot handle barriers well • Largely biased by cell size selected • Interpretation is sensitive to intervals displayed • Time not a factor	• Knowledge of group spread, locational accuracy • Not using 100% points	• Supplement other estimators to look at finer detail of high use areas
*a*-LoCoH & T-LoCoH	• Identifies complex barriers or inaccessible areas • Incorporates time (T-LoCoH) • Robust area estimate with changing sample size or sampling frequency	• Underestimates home range area • No allowance for location uncertainties • Low and variable AUC • User-controlled process in selecting output	• Large dataset • High temporal correlation (T-LoCoH) • Knowledge of natural barriers	• Conservation planning to identify barriers or predator avoidance • Range overlap between groups/species • Core area along sharp barriers
BRB	• Incorporates time • High AUC • Robust area estimates with fix failures • Accounts for location uncertainties • Area robust in variation of parameters selected (*T*_*max*_ and *L*_*min*_)	• Reduced barrier detection as barrier complexity increases • Cannot detect behavioural or biological barriers • Sensitive to decreased sampling frequency	• Species-specific knowledge, locational accuracy • High temporal correlation • Knowledge of natural barriers • At least 200 locations	• Area estimates • Home range for species living along definite habitat edges • Studies with less precise records and more irregular fix success

Grid-cell method (GCM), adaptive local convex hull (*a*-LoCoH), adaptive time local convex hull (T-LoCoH) and biased random bridges (BRB).

There are three main limitations to this study. The first is that it used a single species in one location, and so it is not possible to assess how different environments or home-ranging behaviours might affect the conclusions. Nevertheless, the comparison is valuable alongside other studies comparing home range methods in an increasing diversity of single species (e.g. *Canis familiaris* [32]; *Carcharhinus melanopterus* [71]; *Papio hamadryas ursinus* [44]*; Ursus arctos horribilis* [72]). Furthermore, proboscis monkeys are a good model species for home ranging comparisons as they naturally occur in habitats that have sharp barriers (water-bodies) against which to test the edge-finding ability of different range estimators and, as one of the largest monkey species, are able to wear relatively large GPS collars that can collect high quality data over long periods of time. This made it possible to use subsets of the data to simulate other tracking scenarios.

The second limitation is that the “true” home range of proboscis monkeys, as it is with mammals in general, is unknown, so that whilst we were able to compare different methods and our simulations in terms of their relative performance, there is no way to know the absolute accuracy of the range estimates. Powell and Mitchell [7] suggest that because a mammal’s home range is part of their cognitive map, which is constantly updating, home range estimates can only defined for a specific point in time. Instead, utilisation distribution models can be used predict areas in which the animal is likely to be at a point in time [7]. Simulation studies in the literature have begun to overcome this problem by using artificial tracking data in which the true distribution is known in order to determine the method able to predict the UD most accurately (e.g. [73,74]), but few studies take these simulations a step further to real applications, using data with limitations such as GPS fix failure. Comparing the results from real applications with those from simulations provides greater insight into the differences between methods.

The final limitation is that this not an exhaustive comparison of home range estimators. The number of techniques is increasing each year, all of which have a wide range of parameters that need to be optimised based on the specific study or dataset. It is, however, a realistic application for studies that are restricted in the number of units or animals that can be tracked, or by the size of the study subject and therefore the performance of the GPS tracker. The methods compared here represent both location and movement-based methods, and are some of the key methods developed specifically for GPS data.

### Model performance

Although it is now relied upon less in other ecological fields, GCM is still heavily used in primate studies, as it is computationally simple and easily comparable between studies and sites. Overall, it showed the closest agreement between GPS fixes and the estimated home range, but was the most sensitive to changes in sample size and produced estimates that were distinct from the other three methods (Fig 2). These findings agree with previous studies showing that GCM will produce gross underestimates if the subjects are not followed intensively, making this method unsuitable for studies with longer time intervals between fixes, or random (time) sampling, such as sign surveys [17,18,75]. Using the full data set, GCM and BRB estimates of home range area were similar, but the area of GCM estimates declined substantially in our simulations. However it is important to note that if using 100% of the points, as is standard practice for GCM, the difference in home range area between GCM and BRB would no longer be similar, as the GCM area increased by almost a quarter. The close relationship between sample size and area was also evident with the unchanging AUC value.

The two LoCoH methods were the most robust to changes in sample size for range area and shape, but produced the lowest and most variable AUC values. In the current study, a-LoCoH produced significantly higher AUC values than T-LoCoH, which may be due to the extra parameters of T-LoCoH required to incorporate time. The overall home ranges estimated with *a*-LoCoHs and T-LoCoHs were very similar (Fig 2), which was expected as T-LoCoH was developed as an extension of the location-based *a*-LoCoH [30]. The area estimates were also smaller than the GCM and BRB, which is supported by simulated LoCoH studies showing the hulls created essentially ‘hug’ the data [25,30]. However, this also means that the LoCoH methods are not as strong at modelling spatial uncertainty associated with GPS fixes [30]. They both perform most effectively with large data sets [76]; a-*LoCoH* has been shown to converge on the true range as sample size increases [25].

BRB appeared to show the best overall performance, producing high and robust AUC values, while not showing as much sensitivity to sample size or fix frequency as GCM, which had similarly high AUC values. The positioning of BRB home ranges on the PCoA plot indicates their similarity to those from LoCoH models. Nevertheless, reducing the sampling frequency, and in turn increasing *T*_*max*_, results in a greater degree of smoothing and larger predicted areas for BRB [32], as observed for our Simulation 1. This was evident in the lower edge density and patchiness, and greater overlap with the river. The AUC value showed little change, however, suggesting that the model was still appropriate to use with the current data. The irregular time spacing in Simulation 2, mimicking high fix failure rates, had much less effect on the BRB estimates, producing a similar values to the complete data set for all variables. This was probably because there were periods in the data with higher fix frequencies than the 4h intervals in Simulation 1, allowing better predictions of the tracks taken between fixes. Assuming the dataset meets the requirement of a minimum of 200 locations recommended for utilisation distribution models [28,73], our results agree with previous findings that BRB is well-suited for studies with less precise records and more irregular fix success [32], which is often the case for smaller collars or for collars that have less direct exposure to satellites (i.e. terrestrial forest-dwelling animals).

### Barrier detection

The ability to detect or incorporate barriers is an important function for home range estimates, as including inaccessible areas will overestimate the home range area. This is becoming increasingly important in conservation ecology: there has been a dramatic increase in the number of studies addressing fragmentation and therefore increasingly at sites that include a physical barrier, largely due to habitat loss [77–81]. GCM had the weakest performance, as almost entire cells overlapped the river and oxbow lakes. Grid cells in direct contact with barriers have a large bias, as on average half the cell will be under or overestimated [28]. The amount of overlap with a barrier will be influenced by cell size (here 50 x 50 m), which has also been shown to heavily affect the estimated home range area [17,18,82–84]. The choice of 50 m resolution in this study is already finer than in most primate studies (100–500 m; [12,17,18,85–88]), so the problems of barrier overlap demonstrated here should be relatively conservative.

LoCoH methods on the other hand, were designed to detect hard barriers or areas that seem inaccessible [89]. This property was apparent in the current study, with LoCoH showing consistently the lowest overlap with the river and oxbow lakes (Table 2). LoCoH is capable of identifying sharp and complex boundaries within a few meters, even if the animal is moving along that boundary, as long as the points are taken at a frequent enough rate that corresponds with the movement rates of the species in question [25,30]. Having fewer spatially and temporally auto-correlated points reduces the model’s ability to detect important pathways taken by animals within their home range [90]. Consequently, T-LoCoH works most efficiently with a large dataset with high temporal correlation [30]. The major strength of LoCoH in detecting barriers, such as river edges, can also be its weakness, resulting in the exclusion of areas that are actually used [44].

BRB does not have the same inherent ability to detect barriers as do the LoCoH methods [28], and in order to incorporate barriers in the model, there needs to be *a priori* knowledge of them. Therefore, unlike LoCoH, BRB cannot identify non-geographic barriers, such as group territorial barriers or predator avoidance. The mathematical requirements necessary to implement the barrier also result in some limitations to their use [28], as the requirements can be difficult to satisfy when barriers are complex or have sharp and tight bends, as observed in several sections of the river in this study. The barrier requirements became increasingly difficult to satisfy as *Tmax* increased; when the bend of a river was narrower than a distance of 3*h_*min*_, a simpler boundary had to be used, which subsequently could not include the areas along the river within the sharpest bends, resulting in an increase in the percentage of the home range extending in the river. However, provided the data set has relatively frequent fixes and the barriers are well known (as with the river here), the integrated barrier function performs comparatively well.

### Practical considerations

All of the home range estimator methods considered here require choices to be made for one or more model parameters. For GCM, only the grid cell size needs to be chosen, but as discussed above, this choice can greatly affect the estimated home range area. Often there is little justification given as to the value selected. If GCM is used, biologically based information, such as typical group spread, as well as locational accuracy (i.e. GPS error) needs to be carefully considered in the selection of cell size prior to analysis. Smaller cell sizes may also be better at demarcating areas of importance, and it has been suggested that GCM could be useful when examining habitat suitability and identifying important areas for resource selection [18,91–93].

The LoCoH methods involve selecting an *a*-value directly from the output that visibly looks best to the user. Getz et al. [25] described the standard method of initially selecting the parameter values using the maximum distance between points, then using the “minimum spurious hole covering” rule to refine the parameter based on *a priori* knowledge of the area. Although of the three parameter options (radius, nearest neighbours or adaptive), adaptive is the least sensitive to changes in the parameter value selected [25,30,76], the final selection falls down to the user, to decide, based on visual aids, which value creates the most suitable looking isopleths [30,32], potentially adding bias. Furthermore, LoCoH tends to over-fit the data, resulting in irregular and concave boundaries [44], which was supported in this study by the higher levels of boundary complexity than BRB. Over-fitting may result in an underestimation of home range area by excluding areas in which the animal actually goes [18,44]. LoCoH ‘hugs’ the data [30], and therefore by not providing any buffer around the fix, any surrounding habitat that may be critical for the species is excluded [18]. Consequently, LoCoH does not allow for any location uncertainties around the fix (cf. kernel based methods [29]).

Compared to many location-based kernel density estimates, the parameters chosen for BRB are more intuitive. BRB uses species-specific knowledge as well as the information regarding the precision of the locational data [28], but does not have the same user-defined bias that the LoCoH methods have in looking at the resulting range estimate and making it fit the expected shape. Furthermore, previous studies have shown that adjusting the values of two of the three BRB parameters (*T*_*max*_ and *L*_*min*_) appears to have little effect on isopleth area and shape [32]. In studies applying BRB, a balance will have to be made in selecting a smoothing value that is representative of the GPS data itself, incorporating the resolution of a habitat map, and one that allows for implementing barriers. For species that use definite habitat edges, such as proboscis monkeys, neglecting barriers in the home range analysis could result in an important source of error.

## Conclusions and recommendations

The selection of a home range estimator needs to consider a combination of the underlying research question and information already known about the species and its environment, to determine the most suitable method [8]. Our study of several popular home range estimators revealed some clear differences in performance among the methods. Although GCMs produced the highest and most consistent AUC values, GCM performed worst at barrier detection, generated highly fragmented home range estimates and was the most sensitive method to sample size/sampling frequency. Despite being commonly used in primatology, GCM is not recommended for determining home range boundaries, especially when animals cannot be followed intensively [18], or for a species that use areas with hard barriers, such as proboscis monkeys, who spend a large proportion of the time along water edges. However, GCM may be useful in conjunction with other methods as a simple way to identity areas of importance within the range boundary i.e. as a simple way of estimating the UD. *a*-LoCoH and T-LoCoH were the most robust models to variations in sample size and fix frequency, but had the lowest AUC values and the most variation in AUC values for the simulations. They tended to underestimate the range area, and therefore may not be suitable when looking to conserve an area for a species. Between the location-based and movement-based LoCoH methods, *a*-LoCoH only slightly outperformed T-LoCoH in terms of AUC values as the extra parameterisation in T-LoCoH (scaling factor) However, the incorporation of time in T-LoCoH makes it more biologically relevant in utilisation distribution modelling, and therefore is preferred over the location-based method if the dataset has frequent and regular GPS fixes. The inclusion of time (T-LoCoH and BRB) allows for a more dynamic approach of UDs by further analysis into how often an area is visited, the time spent in those area and the time between visits [30,59]. The additional information that the movement-based methods provide can therefore shed more light on the habitat requirements of an animal, particularly when it comes to conservation planning. Moreover, although LoCoH may not be the most effective method for determining the total area an animal requires, it can also be useful for conservation planning by detecting unused areas within a range or potential restrictions to movement, such as anthropogenic barriers or avoidance of predators [18,34,44], identifying range overlap between species or groups [34,90], or for identifying core ranges along sharp boundaries [89].

With the increasing fragmentation of habitats across the globe, incorporating boundaries in home range analysis is becoming more relevant in more studies. With the inclusion of the barrier feature, BRB seems to be the most suitable overall method for determining the home range of an animal with relatively frequent points, and identifying pathways or routes that are important in the connectivity of an animal’s ranging behaviour. However, this does assume that the relevant barriers are known in advance (e.g. the river in the current study). Where the nature of barriers is uncertain *a priori*, or could follow complex landscape features, or when fixes are at a relatively low frequency, LoCoH methods could complement BRB.

Using the most suitable model, or combination of models, it is possible to understand more fully the patterns, causes, and potential consequences that disturbances could have on an animal, which can then be used to assist in the management and restoration of degraded landscapes [13]. Proboscis monkey ranging behaviour is poorly known, with only two previous estimates, both of which were limited to a single group [43,55]. Using 100% GCM, our home range estimate averaged 108 ha (41–217 ha), which compares to previous estimates using the same method, of 138.3 ha [43] to 220.50 ha [55]. By using GPS collars on multiple proboscis monkey groups, this study showed that BRB was the best-performing HR estimator according to the parameters defined. As GCM tends to over-estimate home range size (as discussed above), the value of home range size of proboscis monkeys in a riparian habitat is smaller using BRB, ranging from 24 to 165 ha, with a mean of 80.89 ha, and therefore should be the most representative estimates of proboscis monkey range to date. Further work using BRB will allow the movement patterns and habitat use within the home ranges to be quantified, alongside the factors affecting the selected range size and variation between the different ranges, contributing further towards the conservation of this endangered primate species.

## Supporting information

S1 Table Number of points used for home range estimates for the complete model. Simulation 1 which mimicked low fix rate (every 4 hours), and Simulation 2 mimicked fix failures.
(PDF)

S2 Table **A**. **Complete model parameters used for adaptive local convex hull (a-LoCoH) and adaptive time local convex hull (T-LoCoH)**
Max. distance is the maximum distance between fixes, and is used as the starting point for determining the *a*-value.
**B**. **Simulation model parameters used for adaptive local convex hull (a-LoCoH) and adaptive time local convex hull (T-LoCoH)**
(PDF)

S3 Table Summary of complete models (90% & 50%). Grid-cell method (GCM), adaptive local convex hull (*a*-LoCoH), adaptive time local convex hull (T-LoCoH) and biased random bridges (BRB).
a,b,c Pair-wise results from Tukey test; results significantly different from another (p<0.05) are indicated by a different letter, those with the same letter showed no significant difference. *Chi-square values for GLMM likelihood ratio test: for all tests, df = 3 and p <0.001.
(PDF)

S4 Table Home ranges areas (ha) for each proboscis monkey group using four home range estimators. Overall home range size using grid-cell method (GCM; 100% & 90%), adaptive local convex hull (*a*-LoCoH; 90%), adaptive time local convex hull (T-LoCoH, 90%) and biased random bridges (BRB; 90%); n = number of GPS fixes used.
*Collared females
(PDF)

S5 Table Simulated model home range area (ha) for each collared proboscis monkey using four methods. Overall home range size (90%) using (1) grid-cell method (GCM), (2) adaptive local convex hull (*a*-LoCoH), (2) adaptive time local convex hull (T-LoCoH, 90%) and (4) biased random bridges (BRB); Simulation 1 simulated low fix rate (every 4 hours) and Simulation 2 simulated fix failures.
(PDF)

S6 Table Summary of simulation core range models (50%). Grid-cell method (GCM), adaptive local convex hull (*a*-LoCoH), adaptive time local convex hull (T-LoCoH) and biased random bridges (BRB). Simulation 1 simulated low fix rate (every 4 hours) and Simulation 2 simulated fix failures.
a,b,c Pair-wise results from Tukey test; results significantly different from another (p<0.05) are indicated by a different letter, those with the same letter showed no significant difference; *Chi-square values for GLMM likelihood ratio test: for all tests, df = 11 and p <0.001.
(PDF)
