# Supplementary material for: Evaluating methods for estimating home ranges using GPS collars: A comparison using proboscis monkeys (Nasalis larvatus)
Source: PLoS One. 2017 Mar 31;12(3):e0174891. doi: 10.1371/journal.pone.0174891 (PMC5376085; doi:10.1371/journal.pone.0174891)
Supplement: S1 Table — Simulation 1 which mimicked low fix rate (every 4 hours), and Simulation 2 mimicked fix failures. (PDF) [file pone.0174891.s001.pdf]

S1 Table

Individual	Complete	Simulation 1	Simulation 2
Group 1	5039	1439	3461
Group 2	3175	903	2163
Group 3	2311	665	1547
Group 4	3081	881	2128
Group 5	5037	1446	3440
Group 6	1498	428	1049
Group 7	3309	948	2234
Group 8	5569	1591	3699
Group 9	1805	512	1279
Group 10	2045	585	1379
