# Supplementary material for: Evaluating methods for estimating home ranges using GPS collars: A comparison using proboscis monkeys (Nasalis larvatus)
Source: PLoS One. 2017 Mar 31;12(3):e0174891. doi: 10.1371/journal.pone.0174891 (PMC5376085; doi:10.1371/journal.pone.0174891)
Supplement: S2 Table — A. Complete model parameters used for adaptive local convex hull (a-LoCoH) and adaptive time local convex hull (T-LoCoH) Max. distance is the maximum distance between fixes, and is used as the starting point for determining the a-value. B. Simulation model parameters used for adaptive local convex hull (a-LoCoH) and adaptive time local convex hull (T-LoCoH) (PDF) [file pone.0174891.s002.pdf]

S2A Table

| Individual | Max. Distance (m) | *a*-value (*a*-LoCoH) | *a*-value (T-LoCoH) | *s*-value (T-LoCoH) |
|---|---|---|---|---|
| Group 1 | 3442 | 1620 | 690 | 0.020 |
| Group 2 | 2383 | 1250 | 730 | 0.028 |
| Group 3 | 1898 | 1480 | 580 | 0.015 |
| Group 4 | 2509 | 2500 | 560 | 0.025 |
| Group 5 | 1598 | 1640 | 760 | 0.028 |
| Group 6 | 1577 | 1470 | 750 | 0.048 |
| Group 7 | 2380 | 1150 | 740 | 0.023 |
| Group 8 | 3497 | 1000 | 640 | 0.019 |
| Group 9 | 3760 | 1060 | 1170 | 0.029 |
| Group 10 | 998 | 790 | 630 | 0.070 |

S2B Table

| | *a*-value (*a*-LoCoH) | | *a*-value (T-LoCoH) | | *s*-value (T-LoCoH) | |
|---|---|---|---|---|---|---|
| Individual | Simulation 1 | Simulation 2 | Simulation 1 | Simulation 2 | Simulation 1 | Simulation 2 |
| Group 1 | 3640 | 3170 | 1200 | 830 | 0.041 | 0.020 |
| Group 2 | 1300 | 1340 | 1270 | 870 | 0.043 | 0.024 |
| Group 3 | 1990 | 2840 | 1050 | 760 | 0.035 | 0.038 |
| Group 4 | 1260 | 2200 | 940 | 860 | 0.053 | 0.025 |
| Group 5 | 1940 | 1770 | 1130 | 1190 | 0.054 | 0.033 |
| Group 6 | 1100 | 1230 | 1230 | 1140 | 0.075 | 0.045 |
| Group 7 | 1420 | 1100 | 1260 | 1110 | 0.048 | 0.024 |
| Group 8 | 770 | 1020 | 1320 | 870 | 0.028 | 0.019 |
| Group 9 | 1270 | 1390 | 1300 | 1240 | 0.050 | 0.028 |
| Group 10 | 1390 | 920 | 1000 | 930 | 0.078 | 0.066 |
