# Supplementary material for: Evaluating methods for estimating home ranges using GPS collars: A comparison using proboscis monkeys (Nasalis larvatus)
Source: PLoS One. 2017 Mar 31;12(3):e0174891. doi: 10.1371/journal.pone.0174891 (PMC5376085; doi:10.1371/journal.pone.0174891)
Supplement: S3 Table — Grid-cell method (GCM), adaptive local convex hull (a-LoCoH), adaptive time local convex hull (T-LoCoH) and biased random bridges (BRB). a,b,c Pair-wise results from Tukey test; results significantly different from another (p<0.05) are indicated by a different letter, those with the same letter showed no significant difference. *Chi-square values for GLMM likelihood ratio test: for all tests, df = 3 and p <0.001. (PDF) [file pone.0174891.s003.pdf]

S3 Table

Method	Ave. Area (ha)	Ave. Edge Density (m/ha)	Ave. Patch Count	Area in river (%)	AUC
**90%**: GCM	83.05[a]	202.77[a]	18.90 [a]	5.00 [a]	0.998 [a]
a-LoCoH	61.41[b]	134.79[b]	2.30 [b]	0.33 [b]	0.841 [b]
T-LoCoH	70.51[c]	122.46[b]	1.50 [b]	0.72 [b]	0.807 [c]
BRB	80.89[a]	108.99[b]	5.10 [c]	2.98 [c]	0.969 [d]
Chi-sq value*	31.22	31.84	54.12	46.18	112.92
**50%**: GCM	50.50[A]	272.26[A]	21.80[A]	4.64[A]	-
a-LoCoH	15.71[B]	333.28[A]	5.80[B]	0.55[B]	-
T-LoCoH	24.72[C]	192.32[B]	2.40[C]	0.97[B]	-
BRB	23.20[C]	204.54[B]	5.90[B]	0.90[B]	-
Chi-sq value*		27.30	49.62	36.87	-
