# Supplementary material for: Evaluating methods for estimating home ranges using GPS collars: A comparison using proboscis monkeys (Nasalis larvatus)
Source: PLoS One. 2017 Mar 31;12(3):e0174891. doi: 10.1371/journal.pone.0174891 (PMC5376085; doi:10.1371/journal.pone.0174891)
Supplement: S4 Table — Overall home range size using grid-cell method (GCM; 100% & 90%), adaptive local convex hull (a-LoCoH; 90%), adaptive time local convex hull (T-LoCoH, 90%) and biased random bridges (BRB; 90%); n = number of GPS fixes used. *Collared females (PDF) [file pone.0174891.s004.pdf]

S4 Table.

	n	GCM: 100%	GCM: 90%	*a*-LoCoH	T-LoCoH	BRB
Group 1	5039	217.00	167.25	124.67	144.15	165.49
Group 2	3175	96.75	76.50	48.09	56.67	62.60
Group 3	2311	69.50	55.50	37.96	42.22	49.18
Group 4	3081	121.00	92.25	76.94	79.55	91.35
Group 5	5037	126.50	112.25	76.24	82.62	83.42
Group 6*	1498	85.25	62.00	55.78	60.37	66.95
Group 7*	3309	125.25	87.25	61.33	74.40	92.88
Group 8	5569	62.00	53.25	21.46	31.31	44.89
Group 9*	1805	136.75	89.25	92.07	109.95	127.98
Group 10*	2045	41.25	35.00	19.60	23.91	24.12
Average (± SE)	-	108.13 (15.71)	83.05 (11.80)	61.41 (10.23)	70.51 (11.56)	80.89 (13.21)
