# Supplementary material for: Evaluating methods for estimating home ranges using GPS collars: A comparison using proboscis monkeys (Nasalis larvatus)
Source: PLoS One. 2017 Mar 31;12(3):e0174891. doi: 10.1371/journal.pone.0174891 (PMC5376085; doi:10.1371/journal.pone.0174891)
Supplement: S5 Table — Overall home range size (90%) using (1) grid-cell method (GCM), (2) adaptive local convex hull (a-LoCoH), (2) adaptive time local convex hull (T-LoCoH, 90%) and (4) biased random bridges (BRB); Simulation 1 simulated low fix rate (every 4 hours) and Simulation 2 simulated fix failures. (PDF) [file pone.0174891.s005.pdf]

S5 Table

(1)

Individual	GCM Complete	Simulation 1	Simulation 2
Group 1	167.25	70.00	138.75
Group 2	76.50	38.25	69.25
Group 3	55.50	28.25	49.25
Group 4	92.25	42.50	79.75
Group 5	112.25	74.50	103.50
Group 6	62.00	24.25	51.50
Group 7	87.25	41.00	71.25
Group 8	53.25	37.75	48.25
Group 9	89.25	27.50	67.75
Group 10	35.00	23.25	32.25

(2)

Individual	*a*-LoCoH Complete	Simulation 1	Simulation 2
Group 1	124.67	128.60	133.17
Group 2	48.09	44.69	47.48
Group 3	37.96	34.55	41.24
Group 4	76.94	74.86	74.74
Group 5	76.24	75.95	78.00
Group 6	55.78	47.73	50.85
Group 7	61.33	59.40	61.46
Group 8	21.46	19.99	20.96
Group 9	92.07	85.87	95.22
Group 10	19.60	18.58	19.97

(3)

Individual	T-LoCoH Complete	Simulation 1	Simulation 2
Group 1	144.15	156.66	146.67
Group 2	56.67	56.61	58.90
Group 3	42.22	42.91	44.18
Group 4	79.55	83.29	87.17
Group 5	82.62	84.86	86.59
Group 6	60.37	64.02	62.84
Group 7	74.40	78.55	80.66
Group 8	31.31	42.55	32.71
Group 9	109.95	105.51	104.27
Group 10	23.91	22.83	25.28

(4)

Individual	BRB Complete	Simulation 1	Simulation 2
Group 1	165.49	227.55	164.02
Group 2	62.60	89.10	62.99
Group 3	49.18	67.83	49.52
Group 4	91.35	119.58	90.22
Group 5	83.42	100.53	83.99
Group 6	66.95	80.07	66.24
Group 7	92.88	190.64	95.71
Group 8	44.89	127.36	52.36
Group 9	127.98	188.44	123.47
Group 10	24.12	34.21	23.96
