# Supplementary material for: Evaluating methods for estimating home ranges using GPS collars: A comparison using proboscis monkeys (Nasalis larvatus)
Source: PLoS One. 2017 Mar 31;12(3):e0174891. doi: 10.1371/journal.pone.0174891 (PMC5376085; doi:10.1371/journal.pone.0174891)
Supplement: S6 Table — Grid-cell method (GCM), adaptive local convex hull (a-LoCoH), adaptive time local convex hull (T-LoCoH) and biased random bridges (BRB). Simulation 1 simulated low fix rate (every 4 hours) and Simulation 2 simulated fix failures. a,b,c Pair-wise results from Tukey test; results significantly different from another (p<0.05) are indicated by a different letter, those with the same letter showed no significant difference; *Chi-square values for GLMM likelihood ratio test: for all tests, df = 11 and p <0.001. (PDF) [file pone.0174891.s006.pdf]

S6 Table

Simulation	Ave. Area (ha)	Ave. Edge Density (m/ha)	Ave. Patch Count	Area in river (%)	Point Inclusion (%)
GCM: Complete	*50.50*[a]	*272.26*[a]	*21.80*	*4.64*	*84.34*[a]
Simulation 1	27.00[b]	468.10[b]	26.60	6.48	74.08[b]
Simulation 2	39.95[c]	320.88[a]	25.30	4.37	78.87[a,b]
a-LoCoH: Complete	*15.71*	*333.28*	*5.80*	*0.55*	*49.49*
Simulation 1	13.65	294.19	3.90	0.87	47.83
Simulation 2	16.12	300.95	5.30	0.47	49.07
T-LoCoH: Complete	*24.72*	*192.32*	*2.40*	*0.97*	*51.43*
Simulation 1	27.11	143.99	1.60	1.21	51.72
Simulation 2	26.80	153.15	1.80	0.76	51.04
BRB: Complete	*23.20*[a]	*204.54*[a]	*5.90*[a]	*0.90*	*56.61*
Simulation 1	31.01[b]	114.97[b]	2.30[b]	2.29	58.68
Simulation 2	23.00[a]	208.70[a]	6.70[a]	0.82	56.36
Chi-sq value*	219.49	169.36	188.67	105.19	270.05
